# Does Neighborhood Social Capital Longitudinally Affect the Nutritional Status of School-Aged Children? Evidence from China

**DOI:** 10.3390/nu15030633

**Published:** 2023-01-26

**Authors:** Lijuan Gu, Linsheng Yang, Hairong Li

**Affiliations:** 1 Key Laboratory of Land Surface Pattern and Simulation, Institute of Geographic Sciences and Natural Resources Research, Chinese Academy of Sciences, Beijing 100101, China; 2 College of Resources and Environment, University of Chinese Academy of Sciences, Beijing 100101, China

**Keywords:** nutritional status, social capital, neighborhood, children, longitudinal analysis, China

## Abstract

Previous research linking social capital to child nutritional status primarily constitutes cross-sectional studies. To investigate whether a longitudinal relationship exists, by conducting fixed-effects analyses with 16,977 repeatedly measured observations of 6193 children from the 2012, 2014, 2016, and 2018 China Family Panel Studies, this study explored the longitudinal effects of neighborhood participation, bonding trust, and bridging trust on the BMI-for-age z-score (BAZ) and BMI categories of school-aged children, differentiating between urban and rural residence. We found an increasing average BAZ, a decreasing prevalence of underweight, an increasing prevalence of overweight/obesity, and a reducing urban/rural gap in nutritional status. The levels of social capital components descended faster in the urban area. Bonding trust was predictive of a lower BAZ, a higher likelihood of being underweight, and a lower likelihood of being overweight/obese. Bridging trust was predictive of a higher BAZ. The longitudinal effects of bonding trust were significant among only the rural children. Our findings indicate that neighborhood social capital may impose causal impacts on the nutritional status of children. To effectively improve child nutritional status, a more empathetic governmental approach that promotes a more supportive distal social environment is needed.

## 1. Introduction

Effectively tackling childhood obesity, one of the major global public health challenges in the 21st century, is urgent, cost-effective, and necessary [1,2]. Although obesity has been biologically ascribed to energy surplus caused by mainly individual factors such as diet, physical activity and genetic predisposition [3], the effectiveness of prevention efforts targeting primarily individual behaviors has been proven to be moderate [4,5]. Given that genetic makeup “loads the gun” and environment “pulls the trigger” [6], the marked rise in obesity reversely mirrors the changes in our environments and the way we live [7], and individual behaviors are largely shaped by distal surrounding environments [3]. Therefore, in understanding the determinants of nutritional status, a socio-environmental perspective underscoring simultaneously the relevance of individual characteristics and nested contexts is widely advocated [7,8]. Among the environmental determinants of health outcomes outside of nutritional status, because there are broader social contexts involved in diet quality and exercise frequency [9], it is argued that the influence of social environmental factors is fundamental [9,10]. Nevertheless, prior socioecological studies concerning nutritional status have mainly concentrated on built environmental components such as obesogens [11], air pollution [12], intersection density [13], and food outlets [14].

The neighborhood is one of the most proximate environmental contexts within which child development happens. The characteristics of the neighborhood are crucial in the socioecological studies of health [15]. Being a major social determinant of health [16], social capital and its associations with nutritional status can provide viable thinking in expanding our understanding of the social patterning of child obesity [9]. Summarizing extant studies linking neighborhood social capital to health, there are at least three possible mechanisms through which it may impact childhood nutritional status [17]. First, the presence of mutual trust and solidarity among neighbors can facilitate collective action on behalf of the common good. For example, advocating for public facilities is in favor of a higher possibility for children to exercise and, therefore, maintain a healthy weight. Second, informal social control within the neighborhood represents a capacity to legitimate health-related behaviors. Living in neighborhoods with prevalent values of minimizing sugar and trans-fat intake is useful in healthy eating among children and then further reducing overweight and obesity [7]. Lastly, neighborhood bonding social capital, namely, the associational ties and level of trustiness, is believed to facilitate better health by buffering stress [9], which may be related to abnormal eating behaviors, sedentary lifestyles, and adverse metabolic changes, which are high-risk factors for overweight and obesity [18].

Research on childhood nutritional status with the components of neighborhood social capital as key predictors is still in its infancy. In 2010, there were four studies linking social capital to nutritional status. In 2018, 22 observational studies investigated neighborhood social capital as a predictor of nutritional status. Among these studies, seven were on children [7]. All studies were from the developed contexts, neighborhood social participation, and neighborhood trust were the most frequently used components, and they arrived at inconclusive findings [7]. Although it is argued that distinguishing from bonding social capital to bridging social capital is useful in understanding those occasionally-reported conflicting findings [17], to the best of our knowledge, studies working on the socio-environmental determinants of childhood nutritional status adopting this strategy are rare. Furthermore, although cohort studies are widely recommended as a critical tool in reducing unobserved heterogeneity and assessing causality [7,19,20], several prior studies conducting longitudinal analyses were on the nutritional status of adult groups [19,21].

The association between neighborhood social capital and nutritional status is context-dependent [7]. Compared to high-income regions, low- and middle-income regions have undergone the most rapid increase in childhood obesity in the past years and may bear a dual burden of undernutrition and over-nutrition [22]. China, as an example, is facing a marked increase in overweight and obese children, with clear urban–rural geographical distinctions in both obesity prevalence and socioeconomic development. On the one hand, according to the latest national estimates, roughly one in five Chinese children is overweight or obese [23]. Although children from the urban China were previously reported to have a higher prevalence of overweight and obesity [22], it is argued that the more recent increase in the occurrence is greater among the rural children [3], and a reversed pattern with higher occurrence in overweight and obesity among the rural children in some developed regions has emerged [3]. On the other hand, China’s unique urban-rural dual structure has been a major social stratification mechanism underpinning its large urban–rural distinctions in health, health-related behaviors, social welfare, and interpersonal connectedness [16]. In rural China, where population flow is slow, there is a close-knitted social connection amongst extended kinship networks and village collectives. Comparatively, with the rapid socioeconomic development and the accelerated life pace, the traditional reliance on in-group connections has diminished in urban China, and urban residents are accustomed to rules, contracts, and regulations [24]. Moreover, compared to rural villagers, urban dwellers have broader chances for social participation, which, in the Chinese context, hinges heavily on the mutual needs of the country and the associations, and which is closely related to socioeconomic status [16]. Given the varying social processes that urban and rural children undergo, the urban–rural distinctions in the neighborhood socio-environmental determinants of childhood nutritional status are highly possible.

By applying fixed-effects analyses to the nationally representative panel survey data of Chinese children aged 6–16 from 2012 to 2018, we aim at an enhanced understanding of the longitudinal relationships between neighborhood social capital and child nutritional status. In our study, by distinguishing between bonding social capital and bridging social capital, an examination of the potentially mixed effects of neighborhood social capital components is enabled. In addition, by differentiating between the urban and rural children, we are able to explore the potentially contextual differences in the socio-environmental determinants of child nutritional status.

## 2. Materials and Methods

### 2.1. Study Design

The nationwide longitudinal social survey project, China Family Panel Studies (CFPS), which collects multiple-level information (i.e., individual, family, and neighborhood) of school-aged children aged between 6 and 16 years at multiple waves, provided the original data. To enable inter-wave consistency of relevant measures, databases from 2012, 2014, 2016, and 2018 were used. In addition to indicators of neighborhood social capital, indicators of family social capital, family and neighborhood economic status, parent-related socioeconomic, and demographic information are also included. By conducting longitudinal analyses with repeatedly measured observations, unobserved heterogeneity in weight status caused by idiosyncratic personal factors is minimized, and we are closer to a causal impact on child nutritional status aroused by changing neighborhood social capital components. Moreover, to delve into the potentially varying associations between neighborhood social capital components and child nutritional status under different socioeconomic strata, we further stratified our analyses by urban/rural residence.

### 2.2. Data

CFPS is a nationally representative longitudinal survey conducted every 2 years since 2010 (http://www.isss.pku.edu.cn accessed on 6 July 2019). The research value of CFPS is assured through strict quality control in the initial conceptual design, survey techniques, interviewing processing, and data processing [25]. To acquire representative samples, CFPS implemented probability proportional-to-size sampling with multistage stratification and used three stages to obtain subsampling. In the first and second stages, administrative districts/counties and administrative villages/neighborhoods, which were selected through official administrative division, were the primary- and second-stage sampling units, respectively. In the third stage, households, which were selected according to a systematic selection protocol, were the ultimate sampling unit.

**Figure 1:**
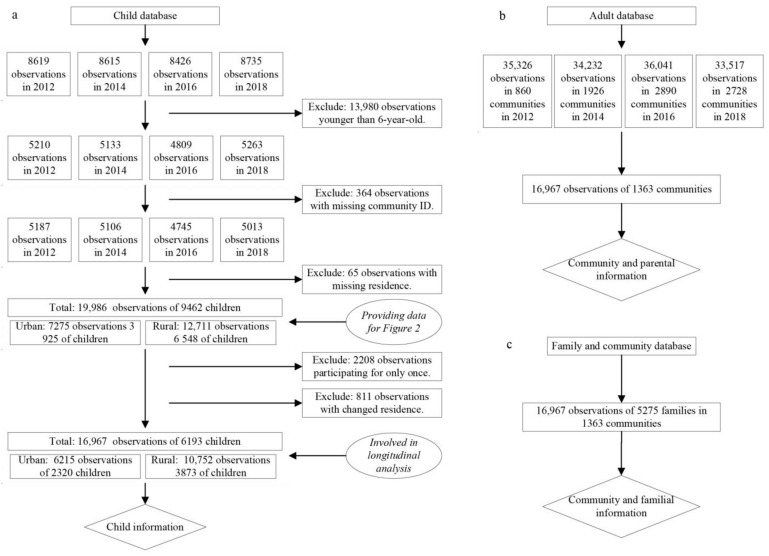
The processing procedures of survey data. (**a**) The processing of child database to provide child information. (**b**) The processing of adult database to provide community and parental information. (**c**) The processing of family and community database to provide community and familial information.

In this three-level designed survey, the community questionnaire focused mainly on neighborhood social welfare, population, facilities, and economic information. At the family level, familial demographic and socioeconomic characteristics were collected. At the individual level, members older than 16 answered adult questionnaires, and members aged 16 or younger answered child questionnaires. Children’s basic information was provided by both their primary guardians at the household and themselves. Given data availability, and considering that the increasing prevalence of overweight and obesity among school-aged children has been an emerging national health issue, school-aged children aged between 6 and 16 years were the focus. The CFPS child database, adult database, family database, and community database represented the original data sources. After several rounds of clearance, we had 19,986 observations of 9462 children (cross-sectional data), whose information was used in general trend analysis. In our longitudinal regression analysis, we had 16,967 observations of 6193 child respondents included (longitudinal data). Among them, 1323 (21.4%) had participated in all waves, 1985 (32.1%) had participated in three waves, and 2885 (46.5%) had participated in two waves. Figure 1 presents a detailed illustration of the procedure of data clearance.

CFPS was conducted according to the guidelines of the Declaration of Helsinki, and it was approved and monitored by the Biomedical Research Ethics Review Committee of Peking University (protocol code IRB00001052-14010, Beijing, China, updated in each survey wave).

### 2.3. Outcome Measures

BMI-for-age z-score (BAZ) and BMI categories were both used as outcome measures. As a continuous absolute measure, BAZ describes nutritional status at the extreme ends of the distribution and enables the examination of gradient relationships. To acquire BAZ, we first used weight in kilograms divided by height in square meters to get the body mass index (BMI). We then calculated the BMI-for-age z-score (BAZ) by standardizing BMI by age and gender according to the WHO Reference 2007 (5–19 years) (www.who.int/growthref, accessed on 16 July 2021). To acquire straightforward comparisons and for ease of interpretation, referencing [3,22], we further used BMI categories including underweight, normal, and overweight/obese as another measure of outcome.

### 2.4. Social Capital Indicators

Neighborhood social capital. Although the term social capital has entered the mainstream of public health since the 1990s, it remains one of the “essentially contested concepts” in social science research [17,26]. There is neither a unified definition nor a standard measure of social capital [16]. However, the emphasis of social capital as the quantity and quality of social relationships stemming from belonging to a group is commonly reported [27,28,29]. The previous literature has primarily used neighborhood members’ overall satisfaction or subjective assessments of neighborhood upkeep, shared values, trust, participation, cohesion, or safety to measure neighborhood social capital [16,26,28], although both the dimensions of social capital components and the measurements of indicators vary across studies. In reference to extant practices and considering data availability, indicators concerning social participation and social trust were used in this study to measure neighborhood social capital. Social participation measured the number of political/cultural/civic/developmental/religious/any other social groups or organizations that the respondents had participated in. Social trust measured how much the respondents trusted their parents, neighbors, people who met for the first time, local government officials, and doctors. Answers ranged from 0 (distrustful) to 10 (very trustworthy). Answers to neighborhood items were provided by all the adult respondents included in the CFPS belonging to each neighborhood. Following extant practice [26], we aggregated the evaluations of within-neighborhood respondents to acquire the levels of neighborhood social capital components. Given the low internal consistency across indicators of trust, factor analysis was further conducted to extract neighborhood bonding and bridging trust.

Family social capital. Likewise, although the term family social capital was systematically introduced by Coleman as early as the 1980s [30], it has neither a unified definition nor a universal measurement. Family ties [26], parent monitoring [31], parent–child interactions, and parental involvement [32,33] have been variably constructed as family social capital components. Drawing heavily from Coleman’s postulation of family social capital as the attention that adults in the family give to children and the relationships between family members (Coleman, 1988), and considering data availability, six indicators concerning the primary caregivers’ involvement with a child’s daily life were used to measure family social capital. Cronbach’s alpha of family social capital indicators is valued at 0.737, indicating good internal consistency. Therefore, the average score was used as a composite measure.

### 2.5. Controls

Children’s age, gender, physical activity and sedentary behaviors, and parental BMI, age, living arrangement, education, marriage, family size, and familial and neighborhood socioeconomic status were listed as controls.

### 2.6. Analytical Strategy

The within-group interrater reliability coefficient ($r_{wg}$) was used to assess the suitability of aggregating individual responses as neighborhood social capital measures [34]. An $r_{wg}$ value of 0.7 or higher indicated high within-group homogeneity [35,36]. The equation of $r_{wg}$ is

$$r_{wg}=1-\frac{s_{x}^{2}}{\sigma_{EU}^{2}},$$

$$\sigma_{EU}^{2}=\frac{\left(A^{2}-1\right)}{12},$$

where $s_{x}^{2}$ is the observed variance across within-neighborhood respondents, A is the number of discrete Likert response alternatives, and $\sigma_{EU}^{2}$ is the variance obtained from a theoretical null distribution.

To net out the influence of unobserved time-constant individual characteristics, we performed longitudinal analyses exploiting repeatedly measured data of 6193 children. Linear fixed-effects regressions were used in the estimation of BAZ, and fixed-effects multinomial logistic regressions were applied in the estimation of BMI categories. To verify robustness, a list of four models was conducted. In Model 1, social capital components were separately included. In Model 2, social capital components were separately included with child age and gender as control variables. In Model 3, social capital components were simultaneously included with child age and gender as control variables. In Model 4, social capital components were simultaneously included with all control variables being considered. To avoid multi-collinearity, centered variance inflation factors (VIFs) for each independent variable throughout models were calculated. Parental age was excluded for being highly correlated with children’s age. Because paternal marital status was closely related to maternal marital status, we kept only the mother’s marriage information in the subsequent regression analyses. All the analyses were stratified by urban/rural residence.

### 2.7. Missing Data

Except for gender and age, all other variables had missing values. The proportion of missing values ranged from 0.68% for family size to 17.61% for paternal educational attainment. Preliminary analyses indicated that the patterns of missing data were not missing completely at random (NMCAR). For example, maternal educational attainment and average family income were independently predictive of missing paternal educational attainment, and parental education was closely related to missing parental BMI. Given the estimation bias of complete case analysis or listwise analysis with NMCAR data, multiple imputation (MI) was applied. To accommodate a mixture of missing patterns (monotone and arbitrary missing) and variable types (continuous and categorical), MI using chained equations (MICE) was chosen. Given the scale of missing data, and considering the efficiency of the estimation process, we first imputed 20 datasets; we then used Rubin’s rules to combine the estimates of coefficients and standard errors of these 20 imputations. We also provided our estimations with 50 imputed datasets for sensitivity analyses in Supplementary Table S2 and Supplementary Table S3.

All analyses were carried out using STATA software, version 15.0 (StataCorp., College Station, TX, USA).

## 3. Results

### 3.1. Descriptive Statistics of Child Participants across Survey Waves

A summary of the cross-sectional information of child participants across survey waves can be found in Table 1. In 2012, the proportions of being overweight/obese were 25.2% and 25.4% among urban and rural child respondents, respectively. This figure exhibited a gradually increasing trend and, in 2018, the proportions of being overweight/obese increased to 28.3% and 27.9% for urban and rural children, respectively. In contrast, the proportion of underweight decreased. For urban children, it decreased from 12.3% in 2012 to 9.7% in 2018. For rural children, it decreased from 13.9% in 2012 to 10.1% in 2018. There were significant differences in the proportions of BMI classifications between urban and rural children in 2012 and 2016, with urban children exhibiting a significantly lower proportion of being underweight (12.3% vs. 13.9%) in 2012, and rural children having a significantly lower proportion of being overweight/obese (26.1% vs. 27.5%) and a significantly higher proportion of being underweight (12.2% vs. 11.2%) in 2016. Comparatively, there were no significant differences in the proportions of BMI categories between urban and rural children in 2014 and 2018.

On average, children were aged 10.53 years according to their cross-sectional information in 2012, 2014, 2016, and 2018. There were more males among the rural respondents (50.6% vs. 48.1%). Rural children spent more hours watching TV, movies, and other videos per week (12.31 vs. 10.49). Urban children exercised more frequently, with 38.3% of respondents exercising four times or more per week. In comparison, this figure was 29.9% among rural children. Fathers’ average BMI was higher among urban children (24.05 vs. 23.26), as also observed for mothers (22.82 vs. 22.53). Similarly, fathers and mothers of urban children were older (39.08 vs. 38.77 for father, and 37.13 vs. 36.86 for mother). Urban children spent more time with both fathers and mothers, and had higher levels of both parental educational attainment and maternal educational attainment. There was no significant difference in paternal marital status between urban and rural children. In contrast, urban children seemed to have a higher proportion of divorced mothers (2.1% vs. 0.6%). Urban children had higher levels of family income, neighborhood economic status, family social capital, and neighborhood participation. In contrast, rural children had larger family sizes, higher neighborhood bonding, and bridging trust.

Table 2 displays the changing characteristics of the total, urban, and rural participants across waves. The longitudinal data of child participants indicated significant differences among urban and rural children in the proportions of BMI categories in 2012 and 2016. In 2012, rural children were significantly higher in the proportion of underweight (14.1% vs. 12.0%). In 2016, rural children were significantly higher in the proportions of both underweight (13.5% vs. 11.6%) and overweight/obesity (27.3% vs. 26.0%). Statistical tests indicated no significant difference in the proportions of BMI categories among urban and rural respondents in 2014 and 2018. Temporally, more than 50% of the respondents had lower BAZ scores, and more than 40% had higher BAZ scores. Compared to urban children, the proportions of rural children who had lower BAZ were significantly higher in both 2014 (57.7% vs. 53.5%) and 2018 (55% vs. 51.3%), and the proportions of children having higher BAZ were significantly lower in both 2014 (42% vs. 46.4%) and 2018 (44.9% vs. 48.4%). It seemed that children were enjoying temporally less family social capital and neighborhood social participation. The difference between urban and rural respondents in the change of family social capital was significant in only 2014, with a higher proportion of rural children having a higher level of family social capital (45.9% vs. 41.5%). The decrease in neighborhood social participation was significantly faster among urban children (59.2% vs. 54.6% in 2014, 58.2% vs. 53.2% in 2016, and 59.6% vs. 53.4% in 2018). In contrast, in comparison to that of urban children, more rural children had higher levels of neighborhood participation (44.6% vs. 38.2% in 2014, 45.6% vs. 38.9 in 2016, and 45.5% vs. 36.2% in 2018). Generally, the majority of children had lower levels of both neighborhood bonding and bridging trust, and higher BMI scores among their parents. Comparatively, the majority of children had unchanged average family income levels.

### 3.2. Temporal Trend of the Nutritional Status of Urban and Rural Children

**Figure 2:**
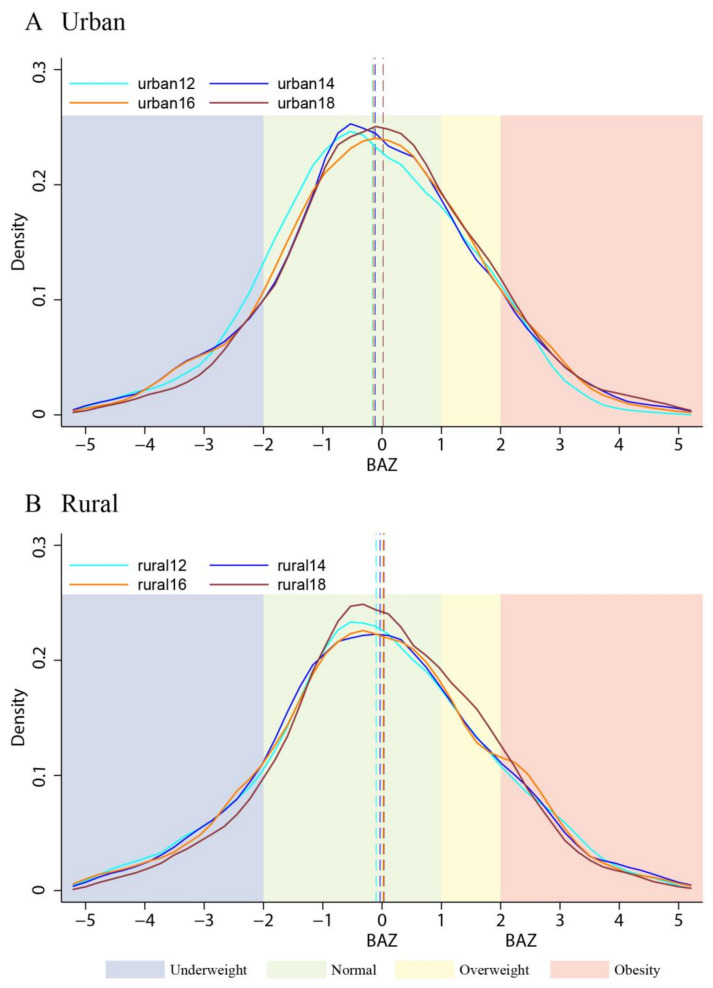
Distributions of BAZ among urban children (**A**) and rural children (**B**) from 2012 to 2018. Note: 1. Curves were plotted with the CFPS cross-sectional data. Epanechnikov kernel density estimates were used with cross-sectional weights adjusted according to the national census (2010, 2020). 2. Dashed vertical lines represent mean values.

Figure 2 depicts a general temporal trend in the distributions of BAZ among the urban and rural children from 2012 to 2018. In both Figure 2A,B, there was a steady shift of the mean values from the left to the right, which implies an increasing trend with time. Areas below the curves with blue shade were smaller with time, which indicates that the prevalence of underweight had decreased. In contrast, areas below the curves with yellow shading were larger, which represents an increase in the prevalence of children being overweight. As shown in Figure 2A,B, compared to that of the urban children, the increase in the accumulated distribution of the normal-weight and overweight children was greater among the rural children. In contrast, the increase in the distribution of children with obesity was more explicit among urban children. As shown in Figure 2 with Table 1, there were temporally narrowing gaps in both BMI categories and BAZ means between urban and rural children.

### 3.3. The Longitudinal Relationships between Social Capital Components and Child BAZ

**Figure 3:**
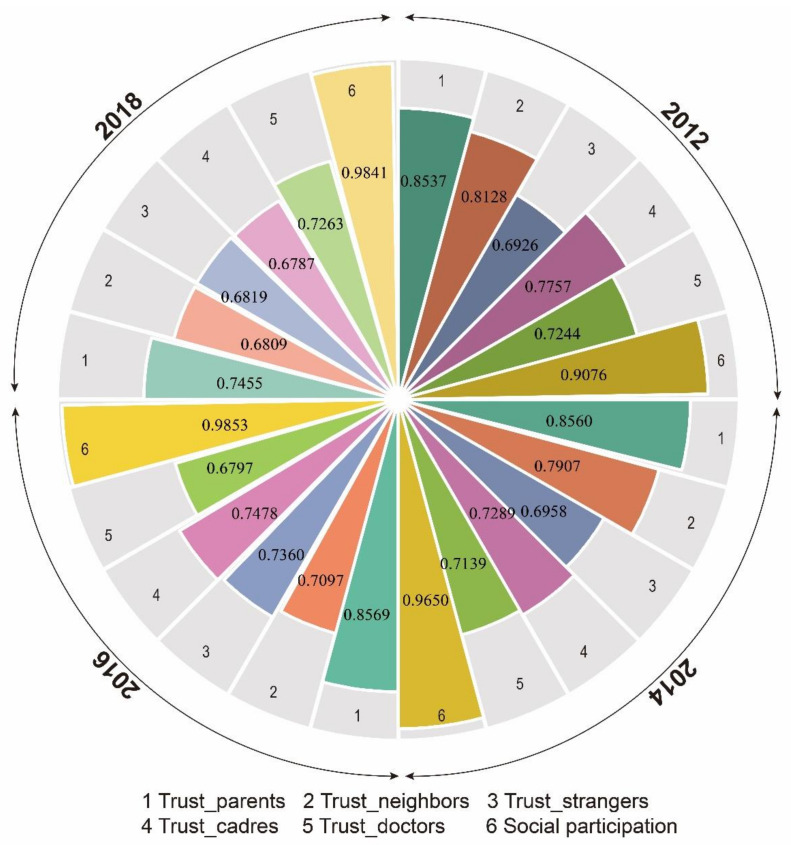
Within-group interrater reliability coefficients of the neighborhood social capital indicators (2012, 2014, 2016, and 2018).

Table 3 lists the survey items of neighborhood social capital components including neighborhood social trust and neighborhood social participation, as well as the survey items of family social capital. Figure 3 depicts the within-group interrater reliability coefficients of the neighborhood social capital indicators in 2012, 2014, 2016, and 2018. The $r_{wg}$ values ranged from 0.6787 for the level of trust in cadres in 2018 to 0.9853 for the level of neighborhood social participation in 2016, indicating acceptable within-neighborhood interrater reliability. Therefore, it is reasonable that we aggregated the within-neighborhood individual data in measuring the level of neighborhood social capital components. Table 4 shows the results of factor analysis with our neighborhood social capital indicators. According to the distribution of factor loadings, neighborhood trust as a component of neighborhood social capital, should be further divided into neighborhood bonding trust, which underscored the degree of trust in parents, neighbors, and doctors, as well as neighborhood bridging trust, which stressed the degree of trust in cadres and strangers.

**Figure 4:**
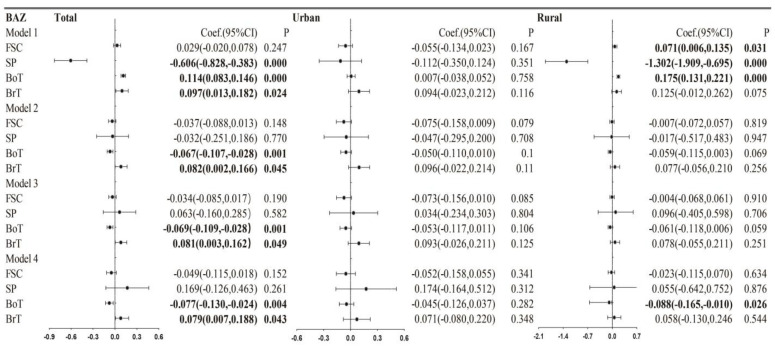
Longitudinal estimations of the relationships between social capital components and BAZ for the total, urban, and rural children. Note: 1. Model 1: Social capital components were separately included. Model 2: Adjusted for children’s age and gender. Social capital components were separately included. Model 3: Adjusted for children’s age and gender. Social capital components were simultaneously included. Model 4: Adjusted for children’s age, gender, physical activity, sedentary behaviors, and parental BMI, age, living arrangement, education, marital status, family size, and familial and neighborhood socioeconomic status. Social capital components were simultaneously included. 2. Estimations were based on 20 imputations.

Figure 4 represents the fixed-effects regression estimates with the BAZ scores for the total, urban and rural children. In Model 1, there was a significantly positive effect of family social capital on the BAZ of rural children, a significantly negative effect of neighborhood social participation on the BAZ scores of the total children and the rural children, significantly positive effects of neighborhood bonding trust among the total children and the rural children, and significantly positive effects of neighborhood bridging trust on BAZ among the total children. In comparison, the impacts of neighborhood components on the BAZ scores of urban children were not significant. With the inclusion of age and gender as controls in Model 2, the coefficients of social capital components among the rural children were no longer significant, the significant coefficients of neighborhood bonding trust and neighborhood bridging trust on BAZ among the total child respondents were retained, and neighborhood bonding trust exerted a marginally significant effect on the BAZ scores of the rural children. With the simultaneous inclusion of social capital components in Model 3, the estimation of coefficients was similar to Model 2. However, with the inclusion of more controls in Model 4, compared to Model 3, the marginally significant effects of neighborhood bonding trust on the BAZ scores of the rural children turned into significant.

Our estimations with 50 imputations indicate similar results (Supplementary Table S2).

### 3.4. The Longitudinal Relationships between Social Capital Components and Child BMI Categories

**Figure 5:**
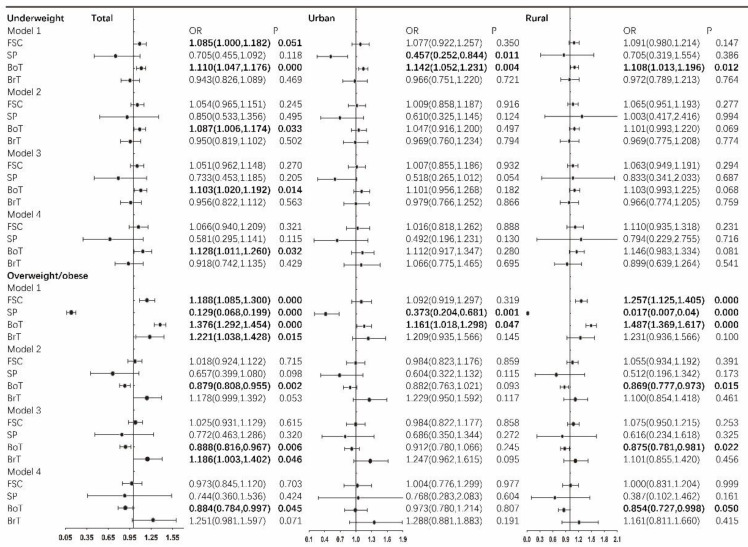
Longitudinal estimations of the odds ratios of being underweight and the odds ratios of being overweight/obese for the total, urban and rural children. Note: 1. Model 1: Social capital components were separately included. Model 2: Adjusted for children’s age and gender. Social capital components were separately included. Model 3: Adjusted for children’s age and gender. Social capital components were simultaneously included. Model 4: Adjusted for children’s age, gender, physical activity, sedentary behaviors, and parental BMI, age, living arrangement, education, marital status, family size, and familial and neighborhood socioeconomic status. Social capital components were simultaneously included. 2. Estimations were based on 20 imputations.

Figure 5 depicts the fixed-effects multinomial logistic estimations of the odds ratios of being underweight and overweight/obese among the total, urban, and rural child respondents. In Model 1, an increase in family social capital was predictive of a higher chance to be underweight among only the total children, and an increase in neighborhood bonding trust was predictive of a higher chance to be underweight among the total, the urban, and the rural children. In contrast, a higher level of neighborhood social participation was predictive of a lower possibility of being underweight among only the urban children. With the control of age and gender in Model 2, merely the significant role of neighborhood bonding trust among the total children remained, and the significant role of neighborhood bonding trust among the rural children turned into marginally significant. With the simultaneous inclusion of social capital components and more control variables in Model 3 and Model 4, odds ratios stayed similar to those of Model 2.

Our estimations with 50 imputations exhibited similar results (Supplementary Table S3).

In the case of overweight/obesity, in Model 1, neighborhood social participation was linked to a lower likelihood to be overweight/obese, and neighborhood bonding trust was linked to a higher likelihood to be overweight/obese; both were significant among the total, the urban and the rural children. In contrast, higher family social capital was predictive of a higher chance to be overweight/obese among the total and the rural children, and higher neighborhood bridging trust was predictive of a higher chance to be overweight/obese among only the total children. With the inclusion of age and gender as control variables in Model 2, the significant role of neighborhood bonding trust among the total children and the rural children remained. However, being opposite to that of Model 1, in Model 2, neighborhood bonding trust was linked to a lower possibility of being overweight/obese. With the inclusion of more variables in Model 3 and Model 4, the role of neighborhood bonding trust in predicting a lower chance of being overweight/obese among the total children and the rural children stayed robust.

## 4. Discussion

This study represents the first longitudinal investigation from the developing context which has adopted national representative data to study the effects of neighborhood social capital components on the nutritious status of urban and rural children. Our findings revealed an increasing average BAZ value, a decreasing proportion of children with underweight, and an increasing proportion of children with overweight/obesity from 2012 to 2018. The urban–rural gap in child nutritional status was reduced, with a greater decrease in the proportion of children with underweight and a greater increase in the proportion of children with overweight in the rural area. The levels of social capital components, including neighborhood participation, neighborhood bonding trust, neighborhood bridging trust, and family social capital, decreased from 2012 to 2018. Compared to that of the rural area, the levels of social capital components in the urban area decreased at a faster pace. Neighborhood bonding trust was longitudinally and robustly related to a lower BAZ, a higher likelihood of being underweight, and a lower likelihood of being overweight/obese. Neighborhood bridging trust was longitudinally and robustly related to a higher BAZ, and marginally significantly related to a higher possibility of being overweight/obese. Moreover, the longitudinal effects of neighborhood bonding trust on BAZ, underweight, and overweight/obesity were significant or marginally significant among only the rural children.

Our findings of the increase in both the BAZ average values and the proportion of children with overweight/obesity echo previous arguments, which declared an improving nutritional status and a rapid increase in the proportion of overweight/obese children in parallel to China’s drastic socioeconomic development [3,22]. Our estimates of the overweight/obesity prevalence among children (25–28%) are higher than the officially reported value of 19%. The varying data sources and different standards that we used may help explain this discrepancy. The official estimate was based on the China Chronic Disease and Nutrition Surveillance survey data from 2015 to 2019, which covered more than 600,000 child respondents. In comparison, ours used the CFPS data in 2012, 2014, 2016, and 2018, with approximately 20,000 child respondents involved. Moreover, for ease of international comparison, we used the WHO standards to define overweight/obesity in this study. In contrast, the official estimate used the Chinese criteria. Given that one previous study, which used a larger scale survey data from 1995 to 2014 covering approximately 1,000,000 participants, reported an occurrence of 20.5% [22], and the temporal trend that Chinese children are getting heavier, our report of the prevalence of overweight/obese children is redeemed to be reasonable.

The shrinking urban–rural disparities in the nutritional status among children are in line with the previous literature [3,22,37]. By longitudinally examining the changing nutritional status of the same child cohort, our study empirically supports the faster increase in the prevalence of overweight/obesity among rural children. There are previous arguments indicating that, because of their better access to energy-dense food and their increased physical inactivity, Chinese children living in the urban area are facing a higher risk of being obese [37,38]. Comparatively, our findings imply that, currently, Chinese rural children may be a more vulnerable group. In addition to the change in dietary habits, the rapid socioeconomic growth in China in recent years has brought about many other consequences. For example, today, rural children are living convergent lifestyles with high accessibility to energy-dose foods. However, compared to their urban counterparts, they have less dietary diversity, and they benefit less from health-related facilities, services, knowledge, and information [22]. These changes in everyday life may reasonably explain the faster increase in overweight/obesity among rural Chinese children.

Our findings of the higher level of neighborhood social participation in urban China, and the higher levels of neighborhood bonding trust and neighborhood bridging trust in rural China verify the arguments of previous studies [16,39]. The close association between social participation and social status in the Chinese context [16,40], and the broader opportunities for social participation for the urban residents have resulted in a higher level of neighborhood social participation in urban China. With China’s unprecedented socioeconomic transition, the traditional in-group connections are gradually decreasing, and contemporary urban dwellers normally handle affairs according to contracts and rules [41]. In contrast, because of a slower flow of population, a well-connected social network based on traditional kinship relationships has been maintained in rural China [42]. As such, compared to that in urban China, the level of neighborhood bonding trust and neighborhood bridging trust in rural China is higher. Our findings of the temporally descending level of both neighborhood participation and neighborhood trust further underscore the necessity and urgency to promote citizen participation and cultivate a sense of belonging in the building of healthy community in China.

Our findings of the robust effect of neighborhood bonding trust in predicting a lower BAZ score and a lower likelihood to be overweight/obese, independent of personal health-related behaviors, personal sociodemographic backgrounds, and family and neighborhood economic levels, are in line with previous studies linking neighborhood social trust to the nutritional status of both adults and children [7,43]. The perception of social trust in a neighborhood may have some overlap with the extent to with neighbors are known and supportive [9], and neighborhood bonding trust represents a primary survival mechanism of “getting by” for residents from disadvantaged neighborhoods (e.g., in a neighborhood with a higher level of bonding trust and enough social support, it is more likely that parents give children more freedom to play outside, even with the presence of disadvantageous physical environment, such as disorders) [17]. Thus, the inverse relationships between neighborhood bonding trust and BAZ, and between neighborhood bonding trust and overweight/obesity are interpretable.

An unexpected finding is that higher neighborhood bonding trust was associated with a higher likelihood of being underweight. To the best of our knowledge, prior studies linking neighborhood social capital components to the chance to be underweight among children are rare. Searching the literature, we propose China’s cultural tastes and preferences toward body shape to provide some reasonable interpretations. China has a cultural conversation about body positivity with many teenagers and young people, especially girls, having an unhealthy obsession with looking skinny [44,45]. According to recent research [46,47], this thin body silhouette is becoming more desirable with time. Because children are more susceptible to trendy norms and peer informal control, children from a highly-bonded neighborhood, who are more likely to be with peers, may have a higher chance of preferring a slender body shape, and of developing some weight-controlling behaviors to achieve an underweight body figure [47]. Echoing previous arguments [7,17], this finding empirically illustrates that the components of social capital may not always be beneficial to health outcomes.

Our results revealed a positive association between neighborhood bridging trust and BAZ. Considering that the relationships between neighborhood bridging trust and BMI categories were not significant, we infer that neighborhood bridging trust played a moderate role in improving BAZ. A higher level of neighborhood bridging trust is indicative of the development of weak ties [17]. It is argued that exposure to a wide range of others from various backgrounds could bring about instrumental support and additional knowledge concerning the formation of healthy behaviors [9]. In our case, for example, a higher level of trust in government officials in the neighborhood may be helpful in guiding the children to respond more actively to China’s national nutrition improvement programs, interventions, and guidance, which aim to increase nutrition intake and improve child health, and which were previously demonstrated to have generated some beneficial impacts [48]. In investigating the relationships between neighborhood social capital components and childhood nutritional status, few previous studies have distinguished between bonding and bridging social capital. By empirically indicating the effect of neighborhood bonding trust in reducing BAZ and lowering the risk to be overweight/obese, and the effect of neighborhood bridging trust in increasing BAZ, our findings are very useful in understanding the inconclusive associations between neighborhood trust and nutritional status among extant literature.

We found clear urban–rural discrepancies in the associations between neighborhood social capital components and childhood nutritional status. The inverse relationship between neighborhood bonding trust and BAZ, the role of neighborhood bonding trust in predicting a higher likelihood of being underweight, and the role of neighborhood bonding trust in predicting a lower likelihood of being overweight/obese were significant among only the rural children. On reflection, the differences in socioeconomic environments between urban and rural China may reasonably explain this phenomenon. As reported in [16], and implied by our data, compared to their rural counterparts, Chinese urban residents generally have a higher level of both educational attainment and economic status. In this circumstance, urban children may rely less on a close neighborhood to acquire health-related knowledge or find a proper site to do outdoor activities. Furthermore, in comparison to the well-knitted close neighborhood based on kinship and village collectives in the rural area, because of the rapid urbanization, massive labor migration, and accelerated life pace in the urban region, urban dwellers are getting accustomed to handling affairs according to rules, contracts, and regulations, and they are experiencing a substantial decline in neighborly interactions, weak local networks, and diminishing neighborly relations [49]. Therefore, it is highly possible that the relationships between neighborhood social capital components and child nutritional status are not significant among urban children. Our findings of the urban–rural differences in the effects of neighborhood social capital components on nutritional status among children empirically support the previous argument, which posits that social contexts matter in exploring the socio-environmental determinants of nutritional status [3,7].

Although neighborhood participation was previously suggested to play a significant role in reducing overweight/obesity [7,19], our study failed to find such an association. Searching the literature, there are other studies finding no significant association between neighborhood social participation and nutritional status [50,51]. The nature of social participation in the Chinese context may help interpret our findings. Unlike the normally voluntary organizational membership in the Western world, organizational participation in China should be formally registered and is closely linked with a superior social status [16]. Because social participation affected the nutritional status through mainly the transmission of healthy norms and behaviors [7,19], with the strict restriction of social status in organizational participation and the small scales of memberships, it is possible that social participation can benefit only a small group of people. Accordingly, the longitudinal relationship between neighborhood social participation and nutritional status among children is not significant.

Unlike the findings of prior studies [9,52], we failed to find a significant association between family social capital and nutritional status. Because of the low fertility rates in contemporary China, children today bear multiple family expectations. As a result, parents involve and invest more in their children’s everyday life and studies. Family involvement in children’s lives can be beneficial to child health. Moreover, family investment in education can be helpful in improving children’s development. However, an over-involvement and an overinvestment can otherwise impose on the children a heavy burden. According to survey data, children’s connectedness to parents was closely related to their feelings of pressure [53]. The literature indicates that, by inducing a physiological response such as higher levels of cortisol or metabolic disruptions, stressful feelings may increase a child’s chance to be overweight/obese [9]. Consequently, family social capital may generate both beneficial and harmful effects on child nutritional status, which may have led to our not significant findings.

This study had some limitations. Although our application of longitudinal analyses with repeatedly measured data brings us closer to a causal relationship between neighborhood social capital components and nutritional status, because reverse causation cannot be totally rejected in cohort analysis, our findings do not necessarily reflect causality. Therefore, to verify the generalization of our main findings, further studies with stronger identification strategies to tease out endogeneity issues are needed. In addition, limited by data availability, we included only the influence of physical activity and sedentary behaviors to minimize the impacts of confounders. However, there are other potential mechanisms, such as dietary behaviors and psychosocial processes, through which neighborhood social capital may affect nutritional status among children. Thus, to achieve a sound understanding of the effects of neighborhood social capital components, future studies working on various mediating pathways are needed. In addition, because of data availability, we merely included social participation, bonding trust, and bridging trust as the measurements of neighborhood social capital. However, a broader coverage of neighborhood components will help gain more insightful findings. Lastly, a small portion of participants were excluded because of their missing neighborhood ID and residential information. Compared to respondents who were included, they were slightly younger, had less sedentary behaviors, and spent less time with their mothers (Supplementary Table S1). To maximize the chances that our results are valid, adjusted weights according to the national census were applied in trend analyses, and multiple imputation using chained equations was used to handle missing data. Nevertheless, instead of being avoided, the potential risk of selection bias could only be reduced.

## 5. Conclusions

This is the first study conducting longitudinal analyses with nationwide repeatedly measured data to get closer to a causal relationship between neighborhood social capital components and nutritional status among children. By stratifying our analyses by urban and rural residence, we delved further into the potential influence of broader social contexts in the socio-environmental determinants of nutritional status. By distinguishing between neighborhood bonding trust and neighborhood bridging trust, we were able to understand the mixed associations between neighborhood social capital components and child nutritional status. Our results indicate a faster increase in the prevalence of overweight/obesity among rural children. The levels of neighborhood social capital components, including participation, bonding trust, and bridging trust, are declining with time. Moreover, the level of neighborhood social capital is declining at a faster pace in urban China. Neighborhood social capital may play a causal effect in affecting child nutritional status. A higher level of neighborhood bonding trust is predictive of a lower BAZ value, a lower likelihood of being overweight/obese, and a higher likelihood of being underweight. In contrast, a higher level of neighborhood bridging trust is predictive of a higher BAZ. There are urban–rural differences in these relationships. The effects of neighborhood bonding trust are significant among only rural children. Considering the fact that the currently insufficient policy attention to wider obesogenic and social environments may have hampered the progress of overweight/obesity control among Chinese children [3], to improve child nutritional status in a more effective way, this study strengthens the evidence base for a more empathetic governmental approach that promotes a more supportive distal social environment.

## Figures and Tables

**Table 1:** Descriptive statistics of the total, urban and rural child participants across survey waves (cross-sectional data, %, mean (CI)).

	Total	Urban	Rural	*p*-Value
**BAZ**	−0.1001 (−0.1003,−0.0999)	−0.1373 (−0.1375,−0.1370)	−0.0615 (−0.0618,−0.0612)	**0.000**
**BMI classification (2012, 2014, 2016, 2018)**				
Underweight	13.4, 12.4, 11.7, 9.9	12.3, 12.3, 11.2, 9.7	13.9, 12.5, 12.2, 10.1	**a**, b, **c**, d
Normal	61.2, 62.5, 61.5, 62.0	62.6, 62.8, 61.3, 62.0	60.7, 62.2, 61.8, 62.0	
Overweight/Obese	25.3, 25.0, 26.8, 28.1	25.2, 24.9, 27.5, 28.3	25.4, 25.3, 26.1, 27.9	
**Age (child)**	10.53 (10.53–10.53)	10.56 (10.56–10.56)	10.50 (10.50–10.50)	0.458
**Gender**				
Male	49.4	48.1	50.6	**0.000**
Female	50.6	51.9	49.4	
**Hours watching TV, movies and other videos/week**	11.178 (11.177–11.179)	10.493 (10.492–10.495)	12.311 (12.308–12.313)	**0.000**
**Physical exercise/week**				
Never	21.7	22.3	21.2	**0.000**
Once/week	6.6	4.9	8.4	
Twice or three times/week	37.5	34.5	40.5	
Four times or more/week	34.1	38.3	29.9	
**Body mass index (father)**	23.57 (23.51–23.62)	24.05 (23.98–23.33)	23.26 (23.19–23.33)	**0.000**
**Body mass index (mother)**	22.64 (22.59–22.70)	22.82 (22.73–22.90)	22.53 (22.46–22.60)	**0.000**
**Age (father)**	38.88 (38.79–38.98)	39.08 (38.94–39.22)	38.77 (38.66,38.89)	**0.002**
**Age (mother)**	36.96 (36.88–37.05)	37.13 (37.00–37.26)	36.86 (36.75–36.98)	**0.004**
**Months living with parents/year (father, mother)**				
Almost never	7.4, 6.2	7.0, 5.1	7.7, 7.3	**e, f**
1 month	9.1, 5.8	6.4, 3.8	11.7, 7.7	
2–4 months	16.9, 11.7	12.0, 7.8	21.4, 15.4	
5–7 months	6.9, 4.6	5.6, 3.4	8.1, 5.7	
8–10 months	4.5, 3.0	3.7, 2.0	5.2, 3.9	
11 months	1.5, 1.3	1.7, 1.2	1.3, 1.4	
Almost the entire year	53.8, 67.4	63.5, 76.8	44.7, 58.6	
**Parental educational attainment (father, mother)**				
No formal education	14.9, 23.1	9.3, 11.5	20.2, 34.0	**g, h**
Primary school	26.5, 26.3	19.3, 20.4	33.2, 31.9	
Middle school	36.3, 32.2	36.6, 36.7	35.9, 28.0	
High school	13.7, 11.3	19.3, 18.3	8.5, 4.8	
College or higher	8.6, 7.1	15.5, 13.2	2.2, 1.3	
**Parental marital status (father, mother)**				
Single	0.4, 0.3	0.3, 0.2	0.5, 0.4	i, **j**
Married/cohabit	96.7, 97.5	96.9, 96.7	96.6, 97.9	
Divorced	2.4, 1.1	2.4, 2.1	2.3, 0.6	
Widowed	0.5, 1.1	0.4, 1.0	0.5, 1.1	
**Quartile—Average Family income**				
1st	28.3	19.8	36.1	**0.000**
2nd	30.7	28.4	32.9	
3rd	25.1	28.9	21.6	
4th	15.9	22.9	9.3	
**Family size**	5.20 (5.18,5.23)	4.78 (4.74,4.82)	5.45 (5.42,5.48)	**0.000**
**Neighborhood economic level (lowest 1–highest 7)**	4.48 (4.40–4.56)	4.93 (4.83–5.03)	4.04 (3.93–4.15)	**0.000**
**Family social capital (standardized)**	3.341 (3.329,3.351)	3.452 (3.434,3.469)	3.272 (3.258,3.286)	**0.000**
**Neighborhood social participation (standardized)**	0.202 (0.200,0205)	0.278 (0.272,0.283)	0.159 (0.157,0.161)	**0.000**
**Neighborhood bonding trust (standardized)**	0.003 (−0.010,0.015)	−0.073 (−0.095,−0.051)	0.045 (0.029,0.060)	**0.000**
**Neighborhood bridging trust (standardized)**	0.139 (0.132,0.146)	0.021 (0.009,0.033)	0.205 (0.197,0.213)	**0.000**

Note: 1. Summarized data were derived on the basis of complete data. Weights were adjusted according to the gender, age and urban/rural distributions of the national census (2010, 2020). 2. a, b, c, and d indicate the significance of comparisons between urban/rural residence in BMI categories in 2012, 2014, 2016, and 2018, respectively (a = 0.000, b = 0.069, c = 0.000, and d = 0.089); e, f, g, h, i, and j indicate the significance of comparisons between urban/rural residence in parent-related information (e = 0.000, f = 0.000, g = 0.000, h = 0.000, i = 0.087, and j = 0.000). Letters and *p*-values in bold imply significant differences.

**Table 2:** Changing descriptive statistics of the total, urban, and rural child participants across survey waves (longitudinal data, *N*, *%*).

	2012			2014			2016			2018		
	Total	Urban	Rural	Total	Urban	Rural	Total	Urban	Rural	Total	Urban	Rural
Participants	*3813*	*886*	*2927*	*4810*	*1903*	*2907*	*4520*	*1816*	*2704*	*3824*	*1610*	*2214*
BMI classification												
Underweight	13.8	12.0	14.1 *^a^*	12.8	11.5	13.1 ^*b*^	12.7	11.6	13.5 ^*c*^	10.1	9.8	10.3 ^*d*^
Normal	61.3	62.9	60.5	61.3	63.3	60.4	60.5	62.3	59.2	61.9	63.1	61.1
O/O	24.9	25.1	25.4	25.9	25.2	26.5	26.8	26.0	27.3	27.9	27.1	28.6
**Change in:** BMI-for-age z-score												
Lower				55.9	53.5	57.7 *^e^*	55.5	55.6	55.3 *^f^*	53.3	51.3	55 *^g^*
Unchanged				0.2	0.1	0.3	0.2	0.2	0.2	0.3	0.4	0.2
Higher				43.9	46.4	42	44.3	44.1	44.4	46.4	48.4	44.9
Family social capital												
Lower				46	48.6	44.1 *^h^*	55.3	56.9	54.2 *^i^*	53.8	53.5	54.1 *^j^*
Unchanged				9.9	9.8	9.9	6.4	6.6	6.3	7.3	8	6.7
Higher				44.1	41.5	45.9	38.3	36.5	39.5	38.9	38.5	39.2
Neighborhood social participation												
Lower				56.4	59.2	54.6 *^k^*	55.3	58.2	53.2 *^l^*	55.9	59.6	53.4 *^m^*
Unchanged				1.5	2.6	0.8	1.2	2.9	1.2	2.4	4.2	1.2
Higher				42	38.2	44.6	43.5	38.9	45.6	41.7	36.2	45.5
Neighborhood bonding trust												
Lower				61.2	64.3	59.2 *^n^*	72.7	68.6	75.4 *^o^*	55.7	60.2	52.7 *^p^*
Unchanged				0.1	0	0.1	0	0	0.1	0.1	0	0.1
Higher				38.7	35.7	40.7	27.2	31.4	24.5	44.3	39.8	47.2
Neighborhood bridging trust												
Lower				76.9	87.5	70.1 *^q^*	86.9	89.8	85.1 *^r^*	71.7	78.2	67.2 *^s^*
Unchanged				0.1	0.1	0.1	0.0	0.0	0.0	0.0	0.0	0.0
Higher				23.0	12.4	29.8	13.1	10.2	14.9	28.3	21.8	37.8
BMI of father												
Lower				35.5	35.0	35.9 *^t^*	30.9	28.1	33.0 *^u^*	38.3	37.0	39.3 *^v^*
Unchanged				13.4	13.6	13.2	26.1	29.2	23.8	13.9	16.4	12.0
Higher				51.1	51.4	50.9	43.0	42.7	43.2	47.7	46.5	48.7
BMI of mother												
Lower				34.9	32.5	36.8 *^w^*	30.2	29.7	30.5 *^x^*	36.0	34.9	36.9 *^y^*
Unchanged				11.6	14.3	9.4	23.7	25.6	22.3	12.5	13.1	12.0
Higher				53.5	53.2	53.8	46.1	44.7	47.3	51.5	52.0	51.0
Average family income												
Lower				26.2	27.7	25.2 *^z^*	32.6	29.3	34.8 *^a’^*	23.4	22.8	23.9 *^b’^*
Unchanged				42.6	43.7	41.9	46.6	47.7	45.9	50.7	50.3	50.9
Higher				31.2	28.6	32.9	20.8	23.0	19.3	25.9	26.9	25.2

Note: 1. O/O overweight and obesity. 2. Letters represent *p*-values, with those in bold indicating significant differences (a = 0.012, b = 0.055, c = 0.031, d = 0.077, e = 0.030, f = 0.988, g = 0.047, h = 0.046, i = 0.283, j = 0.290, k = 0.000, l = 0.000, m = 0.000, n = 0.000, o = 0.000, *p* = 0.000, q = 0.000, r = 0.000, s = 0.000, t = 0.881, u = 0.005, v = 0.005, w = 0.000, x = 0.154, y = 0.460, z = 0.036, a’ = 0.003, and b’ = 0.441). 3. Derived from complete longitudinal data. Panel weights were adjusted according to the gender, age, and urban/rural distributions of the national census data (2010, 2020).

**Table 3:** Survey items of the neighborhood and family social capital components.

Components	Indicators	Response
Neighborhood social trust	How much do you trust your parents?	Ranged from 0 to 10 with 0 meaning distrustful and 10 indicating very trustworthy.
	How much do you trust your neighbors?	
	How much do you trust people you meet for the first time?	
	How much do you trust cadres (the local government official)?	
	How much do you trust doctors?	
Neighborhood social participation	How many political/cultural/civic/developmental/religious/any other social groups or organizations are you in?	From 0 to N
Family social capital	How often do you discuss what happens at school with your child?	1. Never 2. Rarely (once a month) 3. Sometimes (once a week) 4. Often (2–4 times a week) 5. Very often (5–7 times a week)
	How often do you ask the child to finish homework?	
	How often do you check the child’s homework?	
	How often do you restrict or stop the child from watching TV?	
	How often do you know with whom the child is when he/she is not at home?	
	How often do you give up watching TV shows you like to avoid disturbing your child when he/she is studying?	

**Table 4:** Factor loadings of neighborhood social trust indicators.

	Fac 1 (BOT)	Fac 2 (BRT)	Communality
Degree of trust in parents	0.951	−0.068	0.861
Degree of trust in neighbors	0.918	0.138	0.909
Degree of trust in doctors	0.883	0.171	0.809
Degree of trust in cadres	−0.034	0.833	0.695
Degree of trust in strangers	0.166	0.719	0.545

Note: 1. KMO: 0.671; significance of Bartlett’s test: 0.000; total variance explained: 76.41%. 2. BOT: bonding trust; BRT: bridging trust. 3. Initial eigenvalue of BOT: 2.556; initial eigenvalue of BRT: 1.264.

## Data Availability

Restrictions apply to the availability of these data. Data was obtained from the Institute of Social Science Survey (ISSS) of Peking University and are available at http://www.isss.pku.edu.cn/cfps/en/index.htm (accessed on 21 September 2022) with the permission of the ISSS of Peking University.

## Supplementary Materials

The following supporting information can be downloaded at: https://www.mdpi.com/article/10.3390/nu15030633/s1, Table S1: Comparisons of basic descriptive characteristics between participants who were excluded because of missing community ID or/and residence and participants who were included (%, Mean (CI)); Table S2: Longitudinal estimations of the relationships between social capital components and BAZ based on 50 imputations; Table S3: Longitudinal estimations of the odds ratios of being underweight and the odds ratios of being overweight/obese based on 50 imputations.
