# Supplementary material for: Does Neighborhood Social Capital Longitudinally Affect the Nutritional Status of School-Aged Children? Evidence from China
Source: Nutrients. 2023 Jan 26;15(3):633. doi: 10.3390/nu15030633 (PMC9920281; doi:10.3390/nu15030633)
Supplement: Supplementary file 1 [file nutrients-15-00633-s001.zip › nutrients-2013959-supplementary.pdf]

Supplementary Table S1: Comparisons of basic descriptive characteristics between participants who were excluded because of missing community ID or/and residence and participants who were included (%, Mean (CI)).

	Included	Excluded	P value
**BAZ**	-0.1001(-0.1003,-0.0999)	-0.0973(-0.1005,-0.0870)	0.370
**BMI classification (2012, 2014, 2016, 2018)**			
Underweight	13.4	14.6	0.414
Normal	61.2	60.4	
Overweight/Obese	25.3	24.9	
**Age (child)**	10.53(10.53-10.53)	10.01 (9.71-10.30)	**0.001**
**Gender**			
Male	49.4	49.1	0.916
Female	50.6	50.9	
**Hours watching TV, movies and other videos/week**	11.178(11.177-11.179)	9.288(8.334-10.215)	**0.001**
**Physical exercise/week**			
Never	21.7	23.6	0.059
Once/week	6.6	3.1	
Twice or three times/week	37.5	31.0	
Four times or more/week	34.1	42.1	
**Body Mass Index (father)**	23.57(23.51-23.62)	23.60(23.58-23.62)	0.459
**Body Mass Index (mother)**	22.64(22.59-22.70)	22.68(22.63-22.74)	0.444
**Age (father)**	38.88(38.79-38.98)	38.99(38.90-39.11)	0.135
**Age (mother)**	36.96(36.88-37.05)	37.06(36.96-37.16)	0.069
**Months living with parents/year (father, mother)**			
Almost never	7.4, 6.2	8.0, 7.1	a, **b**
1 month	9.1, 5.8	9.2, 6.3	
2-4 months	16.9, 11.7	16.8, 11.8	
5-7 months	6.9, 4.6	6.6, 4.4	
8-10 months	4.5, 3.0	4.7, 2.6	
11 months	1.5, 1.3	1.2, 1.2	
Almost the entire year	53.8, 67.4	53.6, 66.6	
**Parental educational attainment (father, mother)**			
No formal education	14.9, 23.1	14.8, 23.1	c, d
Primary school	26.5, 26.3	26.8, 27.0	
Middle school	36.3, 32.2	36.3, 32.0	
High school	13.7, 11.3	13.6, 11.1	
College or higher	8.6, 7.1	8.5, 6.8	
**Parental marital status (father, mother)**			
Single	0.4, 0.3	0.4, 0.3	e, f
Married/cohabit	96.7, 97.5	96.8, 97.4	
Divorced	2.4, 1.1	2.3, 1.2	
Widowed	0.5, 1.1	0.4, 1.1	
**Quartile_ Average Family income**			
1st	28.3	28.3	0.948
2nd	30.7	30.8	
3rd	25.1	25.1	

4th	15.9	15.7	
**Family size**	5.20(5.18,5.23)	5.19(5.17,5.20)	0.463
**Family social capital (standardized)**	3.341(3.329,3.351)	3.348(3.334,3.369)	0.368

Note: a=0.056, b=0.047, c=0.295, d=0.271, e=0.975, f=0.987

Supplementary Table S2: Longitudinal estimations of the relationships between social capital components and BAZ based on 50 imputations.

**BAZ**	**Total**		**Urban**		**Rural**	
Model 1	Coef.(95%CI)	P	Coef.(95%CI)	P	Coef.(95%CI)	P
FSC	0.029(-0.019,0.077)	0.234	-0.059(-0.137,0.019)	0.138	**0.073(0.008,0.137)**	**0.027**
SP	**-0.606(-0.831,-0.380)**	**0.000**	-0.109(-0.349,0.130)	0.371	**-1.293(-1.889,-0.696)**	**0.000**
BoT	**0.114(0.084,0.145)**	**0.000**	0.008(-0.035,0.052)	0.708	**0.174(0.130,0.219)**	**0.000**
BrT	**0.094(0.008,0.179)**	**0.033**	0.089(-0.027,0.206)	0.132	0.121(-0.019,0.261)	0.089
Model 2						
FSC	-0.037(-0.086,0.011)	0.129	-0.078(-0.160,0.003)	0.058	-0.005(-0.070,0.060)	0.878
SP	-0.032(-0.256,0.192)	0.782	-0.043(-0.292,0.206)	0.737	-0.011(-0.502,0.481)	0.966
BoT	**-0.066(-0.106,-0.027)**	**0.001**	-0.047(-0.108,0.012)	0.117	-0.060(-0.117,0.003)	0.064
BrT	**0.078(0.014,0.163)**	**0.049**	0.092(-0.025,0.208)	0.124	0.073(-0.063,0.210)	0.29
Model 3						
FSC	-0.034(-0.083,0.015)	0.169	-0.077(-0.159,0.004)	0.063	-0.001(-0.066,0.064)	0.971
SP	0.063(-0.166,0.292)	0.593	0.035(-0.234,0.304)	0.797	0.103(-0.392,0.598)	0.682
BoT	**-0.068(-0.108,-0.027)**	**0.001**	-0.051(-0.115,0.013)	0.121	**-0.062(-0.119,0.005)**	**0.034**
BrT	**0.077(0.008,0.162)**	**0.026**	0.088(-0.029,0.205)	0.142	0.075(-0.061,0.2211	0.283
Model 4						
FSC	-0.039(-0.100,0.021)	0.207	-0.017(-0.111,0.076)	0.715	-0.031(-0.116,0.053)	0.471
SP	0.155(-0.116,0.426)	0.262	0.130(-0.170,0.431)	0.394	-0.034(-0.676,0.627)	0.941
BoT	**-0.055(-0.105,-0.006)**	**0.028**	-0.035(-0.109,0.038)	0.347	-0.066(-0.139,0.005)	0.072
BrT	**0.116(0.015,0.216)**	**0.023**	0.065(-0.066,0.197)	0.331	0.115(-0.056,0.288)	0.188

Note: Model 1s: Social capital components were separately included.
Model 2s: Adjusted for children's age and gender. Social capital components were separately included.
Model 3s: Adjusted for children's age and gender. Social capital components were simultaneously included.
Model 4s: Adjusted for children's age, gender, physical activity, sedentary behaviors, and parental BMI, age, living arrangement, education, marital status, family size, and familial and neighborhood socioeconomic status. Social capital components were simultaneously included.

Supplementary Table S3: Longitudinal estimations of the odds ratios of being underweight and the odds ratios of being overweight/obese based on 50 imputations.

**Underweight**	**Total**		**Urban**		**Rural**	
Model 1	OR	P	OR	P	OR	P
FSC	**1.089(1.000,1.187)**	**0.050**	1.076(0.921,1.257)	0.358	1.089(0.974,1.218)	0.133
SP	0.700(0.452,1.085)	0.111	**0.458(0.250,0.838)**	**0.011**	0.690(0.313,1.523)	0.359
BoT	**1.111(1.048,1.177)**	**0.000**	**1.148(1.044,1.264)**	**0.005**	**1.106(1.023,1.197)**	**0.012**
BrT	0.948(0.816,1.102)	0.488	0.961(0.753,1.226)	0.746	0.971(0.780,1.210)	0.794
Model 2						
FSC	1.057(0.967,1.156)	0.224	1.007(0.856,1.185)	0.929	1.069(0.953,1.200)	0.257
SP	0.844(0.530,1.345)	0.475	0.610(0.325,1.148)	0.125	0.978(0.406,2.356)	0.961
BoT	**1.088(1.008,1.174)**	**0.030**	1.049(0.918,1.199)	0.481	1.098(0.990,1.218)	0.076
BrT	0.952(0.819,1.107)	0.522	0.969(0.759,1.237)	0.802	0.966(0.774,1.206)	0.762
Model 3						
FSC	1.054(0.964,1.153)	0.247	1.007(0.855,1.185)	0.935	1.067(0.950,1.198)	0.273
SP	0.729(0.451,1.178)	0.197	0.520(0.265,1.019)	0.057	0.814(0.333,1.989)	0.651
BoT	**1.101(1.018,1.191)**	**0.016**	1.099(0.953,1.267)	0.195	1.102(0.992,1.224)	0.070
BrT	0.958(0.823,1.114)	0.575	0.982(0.769,1.256)	0.887	0.969(0.777,1.210)	0.784
Model 4						
FSC	1.073(0.943,1.221)	0.285	1.016(0.816,1.266)	0.885	1.121(0.939,1.339)	0.206
SP	0.584(0.296,1.153)	0.121	0.496(0.198,1.242)	0.134	0.783(0.226,2.711)	0.699
BoT	**1.126(1.009,1.257)**	**0.033**	1.111(0.917,1.345)	0.283	1.146(0.984,1.334)	0.081
BrT	0.914(0.739,1.131)	0.408	1.059(0.771,1.454)	0.724	0.905(0.643,1.272)	0.565
**Overweight/obese**						
Model 1						
FSC	**1.187(1.084,1.299)**	**0.000**	1.088(0.915,1.295)	0.339	**1.249(1.115,1.399)**	**0.000**
SP	**0.129(0.079,0.212)**	**0.000**	**0.369(0.202,0.675)**	**0.001**	**0.017(0.007,0.043)**	**0.000**
BoT	**1.378(1.296,1.465)**	**0.000**	**1.166(1.050,1.294)**	**0.004**	**1.487(1.369,1.615)**	**0.000**
BrT	**1.229(1.045,1.445)**	**0.013**	1.221(0.945,1.578)	0.126	1.231(0.967,1.568)	0.091
Model 2						
FSC	1.017(0.923,1.120)	0.736	0.982(0.820,1.175)	0.842	1.054(0.933,1.192)	0.398
SP	0.653(0.397,1.075)	0.094	0.598(0.319,1.122)	0.109	0.499(0.190,1.313)	0.159
BoT	**0.879(0.809,0.955)**	**0.002**	0.885(0.763,1.025)	0.103	**0.867(0.774,0.970)**	**0.013**
BrT	1.047(0.998,1.403)	0.056	1.238(0.956,1.604)	0.106	1.103(0.854,1.424)	0.454
Model 3						
FSC	1.024(0.930,1.128)	0.634	0.981(0.819,1.175)	0.835	1.065(0.942,1.204)	0.315
SP	0.768(0.460,1.281)	0.312	0.678(0.346,1.330)	0.258	0.630(0.239,1.659)	0.350

BoT	**0.888(0.815,0.967)**	**0.006**	0.913(0.781,1.067)	0.252	**0.872(0.778,0.978)**	**0.019**
BrT	**1.192(1.008,1.410)**	**0.040**	1.259(0.970,1.634	0.083	1.103(0.853,1.425)	0.455
Model 4						
FSC	0.972(0.845,1.117)	0.685	0.998(0.770,1.295)	0.991	1.001(0.833,1.202)	0.995
SP	0.752(0.364,1.555)	0.443	0.768(0.283,2.086)	0.605	0.384(0.102,1.448)	0.158
BoT	**0.882(0.782,0.995)**	**0.041**	0.970(0.776,1.211)	0.787	**0.853(0.726,0.998)**	**0.049**
BrT	1.252(0.981,1.597)	0.071	1.294(0.885,1.892)	0.184	1.162(0.812,1.662)	0.411

Note: Model 1s: Social capital components were separately included.
Model 2s: Adjusted for children's age and gender. Social capital components were separately included.
Model 3s: Adjusted for children's age and gender. Social capital components were simultaneously included.
Model 4s: Adjusted for children's age, gender, physical activity, sedentary behaviors, and parental BMI, age, living arrangement, education, marital status, family size, and familial and neighborhood socioeconomic status. Social capital components were simultaneously included.
